# Supplementary material for: Effects of Physical Activity on Physical and Mental Health of Older Adults Living in Care Settings: A Systematic Review of Meta-Analyses
Source: Int J Environ Res Public Health. 2023 Jun 26;20(13):6226. doi: 10.3390/ijerph20136226 (PMC10341127; doi:10.3390/ijerph20136226)
Supplement: Supplementary file 1 [file ijerph-20-06226-s001.zip › ijerph-2437349-supplementary.pdf]

Table S1. Search results from the three databases.

	21/10/22	21/10/22	21/10/22	22/03/23
**Keywords**	**Pubmed**	**WoS**	**Cochrane**	**Updating**
‘Physical activity’ AND ‘Nursing homes’ AND ‘older adults’	22	139	3	1
‘Physical exercise’ AND ‘long term care facilities’ AND ‘older adults’	6	17	0	
‘Physical activity’ AND ‘long term care facilities’ AND ‘older adults’	7	17	0	
‘Physical exercise’ AND ‘Nursing homes’ AND ‘older adults’	18	110	2	
‘Physical activity’ AND ‘Nursing homes’ AND ‘elderly’	22	97	2	
‘Physical exercise’ AND ‘long term care facilities’ AND ‘elderly’	6	8	2	
Physical activity’ AND ‘long term care facilities’ AND ‘elderly’	7	13	0	
‘Physical exercise’ AND ‘Nursing homes’ AND ‘elderly’	18	65	0	
	**Total number of items = 582**			

WoS = Web of Sciences

Table S2. Summary of meta-analyses.

References	Participants and Number of Studies	Variables of interest	Interventions
**Alvarez-Barbosa et al. (2020)**	- Total = 557, mean age = 76–85 years and - 10 randomized control trials.	Gait, balance, strength, mobility or quality of life.	Whole-body vibration; program duration: 6–24 weeks; frequency: 2–3 sessions/week; session duration: sessions varied, a total vibration exposure between four and five series or two to six exercises with 5 to 90 sec rest between bouts, with a total vibration exposure between 15 and 60 sec.
**Cao et al. (2018)**	- Total = 551, mean age = 77.8-84.7 years and - Nine randomized control trials.	Falls, TUG (functional mobility), POMA (balance and gait).	Whole-body vibration; weight training and balance exercises, sun-style Tai Chi exercise; program duration: 10 weeks to 12 months; frequency: 1–3/weeks; session duration: 75 sec to 80 min.
**Crocker et al. (2013a)**	- Total = 2379, mean age = 84 years and - Eight randomized control trials and five clusters of randomized control trials.	Activities of daily life (independence score, feeding, dressing, bathing, grooming, toilet use, continence, mobility, transfers, communication, and cognition).	Resistance training + mobility training or balance training or flexibility exercises or ball games or endurance, activities of daily life; program duration: 10 weeks to 52 weeks; frequency: 1–5 sessions/week; session duration: 30 to 150 min.
**Crocker et al. (2013b)**	- Total = 6300, mean age = 69–90 years and - 48 randomized control trials and 19 clusters of randomized control trials.	Activities of daily life: independence in daily activities (Barthel index, functional independence measure, Rivermead mobility index); tests of ability in specific daily activities (TUG, walking time and speed over fixed distance).	Strength training, walking, dancing, ball games, mobility training (walking + wheeling), activities of daily life; program duration: 4 weeks to 12 months; frequency: globally 3 sessions/week; session duration: 9 min to 150 min (median = 45 min).
**Gulka et al. (2019)**	- Total = 30057, mean age = 83 years and - 12 randomized control trials and 24 clusters of randomized control trials.	Number of falls, fallers, and recurrent fallers.	Gait, balance, functional training, strength, resistance, whole body vibration, flexibility, endurance, general physical activity (group or individual; single or multifactorial); program duration: 6 weeks to 15 months; frequency: 2–7 sessions/week; session duration: 15 min to 75 min.
**Kong et al. (2023)**	- Total = 456, mean age = 83 years and - Five randomized control trials and four non-randomized control trials.	Postural balance (Berg balance scale), fall risk, functional mobility (TUG, 6MWT), lower-limb strength (chair stand test), number of falls, and health status.	Otago exercise program; program duration: 3 months to 1 year; frequency: 2–3 sessions/week; session duration: 30 to 60 min.
**Lee and Kim (2017)**	- Total = 5540, mean age = 82.6 years and - 21 randomized trials.	Fall rate; number of fallers (during follow-up).	Balance and strength training; program duration: 4 to 48 weeks; frequency: 1–3 sessions/week; session duration: 6–90 min.

Table S2. Continued.

References	Participants and Number of Studies	Variables of interest	Interventions
**Li et al. (2022)**	- Total = 2594, mean age = 81.8 years and - Eight randomized control trials, five quasi experimental designs, four clusters randomized control trials, two longitudinal designs, one interventional design, and one study for which experimental design is not available.	Depression score (clinical-report scales); reported depression (self-report depression scales).	Mix of aerobic, muscle, and bone strength and stretching/flexibility; Tai chi, yoga; program duration: 4 to 64.5 weeks; frequency: 1 to 7 sessions/week; session duration: 20 to 90 min.
**Schoberer and Breimaier (2019)**	- Total = 3767, mean age = 65–70 years and - 23 randomized control trials.	Number of falls; number of fallers (during follow-up); fall rate; injuries sustained from falls (e.g., hip fracture); fear of falling; quality of life.	Tai chi, balance, strength, whole body vibration, walking, endurance, flexibility, activity training with goal setting, yoga, and mobility; program duration: 4 weeks to 24 months; frequency: 1–5 sessions/week; session duration: 6 to 90 min.
**Silva et al. (2013)**	- Total = 1292, mean age = 83.9 years and - 12 randomized control trials.	Falls and fracture prevention.	Balance, muscle strength + endurance, stretching, gait, mobility, walking; program duration: 3–24 months; frequency: 1–3 sessions/week; time session: 30–90 min.
**Wang and Tian (2021)**	- Total = 2748, mean age = 86 years and - 14 randomized control trials.	Number of fallers.	Whole-body vibration, balance and strength, stretching, goal setting physical activity; program duration: 4 to 48 weeks; frequency: intervention ranged from multiple times a day to 1–2 times a week; session duration: 3 min to 90 min.

TUG = Timed Up and Go test; 6MWT = 6 Minute Walk Test; POMA = Performance Oriented Mobility Assessment
